# Comparing Types of Yoga for Chronic Low Back and Neck Pain in Military Personnel: A Feasibility Randomized Controlled Trial

**DOI:** 10.1177/2164957X221094596

**Published:** 2022-06-16

**Authors:** Erik J Groessl, Danielle Casteel, Symone McKinnon, Adhana McCarthy, Laura Schmalzl, Douglas G Chang, Ian M Fowler, Crystal L Park

**Affiliations:** 1 Herbert Wertheim School of Public Health, University of California San Diego, La Jolla, CA; 2 HSR&D, VA San Diego Healthcare System, San Diego, CA; 3 US Army, 187th Med Student Detachment; 4 College of Science and Integrative Health, Southern California University of Health Sciences, Whittier, CA, USA; 5 Physical Medicine and Rehabilitation, Dept. of Orthopedic Surgery, University of California San Diego, La Jolla, CA; 6 Naval Medical Center San Diego, United States Navy; 7 University of Connecticut, Department of Clinical Psychology, Storrs, CT

**Keywords:** Chronic pain, disability, yoga, military personnel, feasibility

## Abstract

**Background:**

Chronic low back pain (cLBP) and chronic neck pain (cNP) are highly prevalent conditions and common reasons for disability among military personnel. Yoga and other mind-body interventions have been shown to safely decrease pain and disability in persons with cLBP and/or cNP but have not been adequately studied in active duty military personnel. The objective of this study was to examine the feasibility and acceptability of delivering 2 types of yoga (hatha and restorative) to a sample of active-duty military personnel with cLBP/cNP.

**Methods:**

Military personnel with cLBP and/or cNP (n = 49; 59% men) were randomized to either hatha or restorative yoga interventions. Interventions consisted of in-person yoga 1-2x weekly for 12 weeks. Feasibility and acceptability were measured by rates of recruitment, intervention attendance, attrition, adverse events, and satisfaction ratings. Health outcomes including pain and disability were measured at baseline, 12 weeks, and 6 months. Means and effect sizes are presented.

**Results:**

Recruitment was completed ahead of projections. Over 90% of participants agreed or strongly agreed that they enjoyed participation, liked the instructor, and would like to continue yoga. Retention rates were 86% and 80% at 12 week and 6 month assessments, respectively. Intervention attendance was adequate but lower than expected. There were small to moderate reductions in back-pain related disability, pain severity and pain interference, and improvements in quality of life, grip strength, and balance. In general, effects were larger for those who attended at least 50% of intervention classes. Participants with cNP tended to have smaller outcome improvements, but conclusions remain tentative given small sample sizes.

**Conclusions:**

Results demonstrate feasibility for conducting a randomized controlled comparative effectiveness trial of yoga for cLBP and cNP among active duty military personnel. Acceptability was also established. Ongoing work will enhance the intervention for cNP and establish feasibility at another military facility in preparation for a fully-powered comparative effectiveness trial.

ClinicalTrials #NCT03504085; registered April 20, 2018.

Low back pain and neck pain are highly prevalent conditions that become chronic in 20–30% of those afflicted.^1^ Many people with chronic pain also experience decreased functional ability,^2^ lower productivity in work settings,^3,4^ increased psychological symptoms, (depression,^5,6^ anxiety^7,8^), lower sleep quality,^9,10^ and higher health care costs.^11^ Recommended treatment for chronic low back pain (cLBP) and chronic neck pain (cNP) typically begins with medication management and self-care information,^12^ but the limited effectiveness of these approaches and risks associated with pain medication magnifies the need for additional non-pharmacologic treatment options.^13,14^ Of the non-pharmacological approaches reviewed by Chou et al,^15^ none have large effects for cLBP/cNP or are considered superior to others.

Military personnel^16,17^ and veterans^18^ have higher rates of cLBP/cNP than the general US population, and cLBP/cNP are two of the most common reasons for disability among deployed personnel^17^ and the military in general.^19^ cLBP/cNP in military personnel may be harder to treat when co-morbidities such as PTSD^20,21^ are present. Although more research is needed, causal factors for cLBP/cNP in military personnel likely differ from those in the general population, with some data suggesting that overseas deployment, combat experiences, and physically demanding duties increase the risk of developing cLBP/cNP in military personnel.^16,19^ Other context-specific factors including high-stress environments, pressure to perform and advance, and/or stigma may also interfere with efforts to treat and address chronic pain in active-duty military personnel.^22,23^

Yoga is a mind-body intervention that typically involves combinations of physical postures and movement, deep breathing, and focused attention.^24^ Thus, yoga interventions are multifaceted, with documented physical and psychological benefits including both a) increased strength, flexibility, and conditioning through the performance of physical postures, and b) stress reduction/relaxation and improved psychological functioning facilitated by deep breathing exercises, concentration/attention, and cognitive strategies. Strong evidence from a number of large RCTs in community samples indicates that yoga reduces pain and disability among persons with cLBP,^25–27^ and yoga is now recommended as a front-line treatment for cLBP in treatment guidelines.^15^ More recently, an RCT of yoga for cLBP in Veterans Affairs patients found yoga produced similar results among military veterans.^28^ Despite these positive results in military veterans, active-duty military personnel with cLBP/cNP are quite different from VA patients in that study.^29^ They are considerably younger and more active, have different occupational requirements and social needs, and may have different medical needs due to comorbidities, all of which may benefit from tailored treatment strategies.^23,30^

A growing body of research evidence also supports the use of yoga for treating cNP.^31,32^ Although almost all previous studies of yoga for chronic pain focus on a single type of pain condition (osteoarthritis, cLBP, or cNP) a goal of this study was to create and test yoga interventions that can treat more than a single type of chronic pain. This pragmatic approach is warranted because it is unlikely that health organizations can offer separate yoga interventions for each specific pain condition.

The vast majority of research on yoga for improving pain and function in persons with cLBP has involved hatha yoga;^33,34^ yoga that consists of active movement through various physical postures while incorporating aspects of breath work, concentration, and relaxation.^24,35^ However, there is growing evidence that other types of yoga and other mind-body practices may help people with chronic pain even though physical postures and movement are minimally present in them.^36,37^ Yet no previous research had compared preferences for or response to physically active or inactive/relaxing yoga interventions.

Thus, since limited research exists on yoga for chronic pain in active duty military personnel, our objective was to establish the feasibility and acceptability of conducting high-quality research comparing two different types of yoga (active hatha yoga vs restorative yoga) for treating cLBP and/or cNP in active duty military personnel. Comparing types of yoga may provide insight to which types of yoga are best suited for various chronic health conditions.

## Methods

### Design

Active-duty military personnel with cLBP and/or cNP (n = 49 total) were randomized to either active hatha yoga or restorative yoga interventions. Both interventions consisted of in-person yoga instruction 1 to 2 times weekly for 12 weeks, with daily home practice recommended. We chose this option based on results of a prior study,^23^ and in an effort to conduct pragmatic research. The study was conducted by university researchers in collaboration with a large military medical center. The main study goal was to assess the feasibility and acceptability of conducting a full-scale clinical efficacy trial in this context. The main metrics of feasibility and acceptability were rates of recruitment, intervention attendance, attrition from assessments, participant satisfaction ratings, and adverse events. In addition, health outcomes were measured using self-report questionnaires and physical performance measures at baseline, 12 weeks, and 6 months. The target sample size was 50 participants comprised of 2 cohorts of approximately 24-26 participants per cohort, providing 12-13 individuals per intervention in each cohort. The study was approved by the UC San Diego Human Subjects Protection Program in agreement with the US Navy.

### Recruitment

Participants were recruited from January through September of 2018. The majority of participants were recruited at the Naval Medical Center San Diego (NMCSD). Clinicians in Pain Medicine at NMCSD and other clinics notified their patients about the study. Other recruitment methods included word of mouth and flyers posted at NMCSD, cafes, community centers, and public posting boards in areas frequented by military personnel.

### Screening and Enrollment Criteria

Potential participants were pre-screened via telephone to review criteria such as active-duty military status, no recent yoga practice, and willingness to participate in intervention and assessments. Clinical research staff at NMCSD screened participants using specified medical criteria. Exclusion criteria are shown in Supplemental Table 1.

### Baseline Assessment and Randomization

Eligible participants who remained interested were scheduled for an informed consent and baseline assessment appointment. After providing consent and completing the assessment, participants were randomly assigned to either the active hatha yoga or restorative yoga treatment group. Randomization was completed by the project coordinator using a computer program (1:1 ratio, blocks of 25 to balance groups) created by the statistician. Allocation was concealed and only available upon randomization.

### Retention and Attendance

The scientific importance of completing study assessments and attending assigned interventions was discussed with research participants by research staff at time of consent. Retention at assessments was encouraged with phone call reminders about upcoming assessments, especially between the 12 weeks and 6 months assessments. Additional reminder e-mails were provided preceding assessments when phone contact was not successful. Regular attendance at yoga sessions and engaging in yoga home practice were emphasized by the instructor during and after yoga sessions. All participants were contacted by study staff if they inexplicably missed an intervention session to encourage resumption as soon as possible. The methods have been used in prior studies.^28^

Each intervention was offered at 3 different times each week (weekday morning - 7 AM, weekday early evening - 5 pm, weekend mornings – 8 AM) to facilitate attendance. Weekday sessions were held at the NMCSD while the weekend yoga session was held at a nearby community yoga studio. All participants received a free yoga mat for enrolling in the study.

### Active Hatha Yoga intervention

The active yoga intervention consisted of 60 minutes sessions for 12 weeks led by certified) instructors who had 3 + years of experience teaching yoga to military populations with health issues. Participants were asked to attend at least once per week but were allowed to attend a second class if they desired or if they had missed or expected to miss another session. The intervention was based on and similar to a yoga intervention used in prior studies.^38,39^ However, study investigators and yoga experts reviewed the intervention for the current study and created a revised yoga instruction manual to a) increase the pace and vigor of movement for younger, more able active duty participants; b) adapt the practice to address chronic neck pain (cNP).

The resulting yoga intervention retained influences from Viniyoga and Iyengar yoga, which emphasize modifications including the use of props such as straps and blocks to minimize the risk of injury and make poses accessible to participants with varying degrees of functional abilities.^3^ Using the manual as a guide, yoga instructors led participants through a series of 23 yoga poses (32 total variations) at a moderate pace. Yoga sessions began with approximately 5 minutes of instructor-led breathing practices and a brief meditation, followed by seated poses (15-20 minutes), standing poses (10-15 minutes), floor poses (15 minutes), and a supine resting pose (Savasana; 10 minutes). Using a home practice manual, participants were encouraged to practice basic poses at home for 15-20 minutes each non-instruction day, while emphasizing safety.

### Restorative Yoga Intervention

Restorative Yoga is a slow-paced yoga style that emphasizes relaxation and includes very little movement. Like active yoga, the restorative intervention consisted of 1-2x weekly 60 minutes sessions for 12 weeks led by certified, experienced (3 + years) instructors with experience teaching yoga to military populations with health issues. Participants spend most or all of the session in seated or reclining poses, often with their eyes closed. Sessions typically included 5-10 poses total, with slow non-strenuous transitions between poses. Bolsters and blankets were provided for comfort and warmth. The instructor provides instruction and dialogue on breathing techniques, guided imagery, and positive affirmations or suggestions to promote relaxation and healing.

### Measures

Feasibility and acceptability were measured with pre-specified metrics including recruitment rates, attendance, attrition, adverse events, and program satisfaction, all of which were tracked by study staff. Attendance at intervention sessions was tracked by a sign-in sheet verified by the coordinator or instructor each week. Yoga home practice compliance was assessed using summary questions at the 12-week assessment.

#### Feasibility/Acceptability

Recruitment rates, retention/attrition rate at assessments, intervention attendance rates, and adverse events served as the main measures of feasibility and acceptability. Another important initial indicator of feasibility was the time to obtain institutional review board (IRB) approval for this research through the NMCSD and the US Navy. Including IRB approval as a measure of feasibility was written into the grant and approved by NCCIH based on anecdotal evidence suggesting that obtaining IRB approval in military settings can be a time-consuming process and a potential barrier to success for non-military researchers. Program satisfaction ratings provided a final measure of acceptability. Metrics specified in communications to the funding agency that would indicate adequate feasibility to proceed included:

(1). Recruitment of 50 active-duty military personnel in 18 months or less
(2). Retention of at least 75% of all randomized participants at each assessment point
(3). Intervention attendance of at least 50%

Time to obtain IRB approval did not have a specified minimum for feasibility, but up to 12 months was allocated in the study timeline. Program satisfaction ratings and adverse events rates did not have pre-specified levels. Satisfaction ratings were assessed using 10 questions rated on a Likert scale (0 – no satisfaction; 5 – very high satisfaction) used by the investigators in previous and yoga studies. All feasibility and acceptability data were evaluated by an Independent Monitoring Committee consisting of a yoga research expert, a clinical content (cLBP/cNP) physician expert and a biostatistician.

#### Adverse Events Monitoring

Adverse events were primarily assessed through phone calls to any participants who that did not attend at least 1 yoga session in a given week without notifying staff of a conflict. Participants who missed an intervention session without explanation were contacted by phone to encourage future attendance and assess adverse events. In addition, instructors asked those attending each yoga session if they had experienced any significant health problems during the week.

### Health Outcomes

Assessments at baseline, the end of the intervention (12 weeks), and 6 months after baseline consisted primarily of self-report questionnaires followed by 2 physical performance tests administered by trained assessors. Assessors were blinded to intervention condition and participants were reminded to avoid disclosing their intervention assignment to assessors. Participants received a $50 gift card for completing each assessment.

Pain interference with daily function and pain severity were measured with the Brief Pain Inventory (BPI).^40^ The BPI has been validated with cLBP.^41^ The pain interference score is the mean of the 7 interference items, and the pain severity score is the mean of 4 severity items. Scores on each item and thus the total scores range from 0 to 10. It has good reliability (alpha .77 - .91). The PROMIS Pain Intensity is a validated 3-item scale where pain is rated on a 5-point Likert scale.^42^ The items are averaged resulting in a total score ranging from 1.0 to 5.0.

### Disability/Physical Function

The *Roland-Morris Disability Questionnaire (RMDQ)* consists of 24 yes/no questions that ask about limitations experienced for a variety of daily activities (score range 0-24). The scale has been shown to be reliable, is well validated,^43^ and has been used in other yoga studies.^44^ *The Neck Disability Index (NDI)* was used to assess disability related specifically to neck pain. It is a well validate measure.^45^ *Health-related quality of life (HRQOL).* The 12-item Short-form Health Survey (SF12) was derived from the SF-36.^46^ The SF12 physical and mental component scores (range 0-100) have been shown to be similar to the SF36 scales in terms of precision and sensitivity to change. *Fatigue.* The Fatigue Severity Scale (FSS) assesses the impact and severity of fatigue with 9 items. The total score is the mean of the 9 items ranging from 1 = strongly disagree to 7 strongly agree. A score of ≥4.0 constitutes severe fatigue.^47^ The measure has good psychometrics for pain disorders.^47,48^ *Self-efficacy.* Self-efficacy for managing pain reflects confidence in the ability to manage the intensity or impact of cLBP/cNP on daily life. The 6 questions are based on items developed by Lorig et al^49^ and the total score is the mean of the items (range 1-10).* Alcohol Use*. The AUDIT-C is a 3-item alcohol screen that reliably identifies patients who are hazardous drinkers or have alcohol use disorders (Scores range from 0-12).^50^ *Depression.* Derived from the full Center for Epidemiologic Studies Short Depression Scale^51^ (CES-D), the CES-D 10^52^ consists of 10 items on the frequency of mood symptoms. Scores range from 0 to 30, and scores ≥10 indicate elevated symptoms of depression. *Resilience.* The Brief Resilience Scale (BRS) was used to assess the ability to recover from stress or trauma. The measure consists of 6 items and has good reliability and validity (range 0-5).^53^ *Anger.* Developed by Forbes et al,^54^ The Dimensions of Anger Reactions questionnaire consists of 7 items and has demonstrated strong internal reliability and concurrent validity with other existing measures of anger. The total score is a sum of items (range 5-35).

*Grip Strength* was included to facilitate comparisons with previous trials^28,55^ and was measured using two trials for each hand with a hydraulic dynamometer. The average of 2 trials was used for analysis. Test-retest reliability of this measure of grip strength has been shown to be high: r =.88-.92. Good predictive validity of grip strength has been shown for disability and mortality.^56^* Balance.* The Single Leg Stance (SLS) is a commonly used measure of both lower leg strength and balance.^57,58^ The SLS is a timed test in which participants stand on 1 leg at a time for up to 60 seconds, first with both eyes open and then with both eyes closed.

### Statistical Analysis

As a feasibility study, the study was not powered to evaluate hypotheses about group differences. Thus, means and raw data were reported for measures of feasibility. Within-group effect sizes were calculated using Cohen’s d for the change in health outcome means over time, divided by the pooled standard deviation of the 2 data points.^59^ 95% confidence intervals are provided. Modified intent-to-treat analyses were conducted without imputation of missing data.

## Results

The study sample (n = 49) self-identified as 41% Women and 59% Men who were active duty military personnel in the US Navy (n = 40) and US Marine Corps (n = 9). Participants were 37% non-white, including 21% black, 15% Asian, and 2% American Indian or Pacific Islander. Ethnically, 22% self-identified as Hispanic (See Table 1). When comparing characteristics of the sample to data from all US military personnel, women were over-represented (41 vs 17%) while racial and ethnic minority groups were represented in proportions similar to the population.^60^

**Table 1.** Demographics.

	Hatha (n = 24)	Restorative (n = 25)
Age mean (*SD*)	34 (9.1)	32 (8.1)
Gender		
Male	16 (67%)	13 (52%)
Female	8 (33%)	12 (48%)
Other	0	0
Military branch		
Navy	20	20
Marines	4	5
Ethnicity		
Hispanic/latino	3 (12%)	8 (32%)
Not hispanic/latino	21 (88%)	17 (68%)
Race		
White	16 (67%)	15 (60%)
Black or African American	3 (12%)	7 (28%)
Asian	4 (17%)	3 (12%)
American Indian or Alaskan Native	1 (4%)	
Marital status		
Single-never married	3 (13%)	2 (8%)
Cohabitating	1 (4%)	2 (8%)
Married	17 (71%)	16 (64%)
Divorced or separated	3 (12%)	5 (20%)
Education		
High school or equivalent	4 (17%)	9 (36%)
Technical school or college	9 (4%)	8 (32%)
Bachelor’s degree	9 (37%)	4 (16%)
Graduate degree	2 (8%)	4 (16%)
Household income		
$20K-$39K	4 (17%)	3 (12%)
$40K-$59K	5 (21%)	7 (28%)
$60K-$79K	5 (21%)	5 (20%)
$80K-$99K	2 (8%)	5 (20%)
$100K-$249K	8 (33%)	5 (20%)
Chronic back and neck pain		
CLBP	13 (54%)	13 (52%)
CNP	2 (8%)	4 (16%)
Both	9 (38%)	8 (32%)

### Feasibility and Acceptability

An initial feasibility measure was time to obtain an approved IRB (human subjects research) protocol at both the hosting university and a nearby naval medical center. The study allocated 6-12 months for completion and the process with full approval was obtained in 11 months.

#### Recruitment

**Figure 1.**
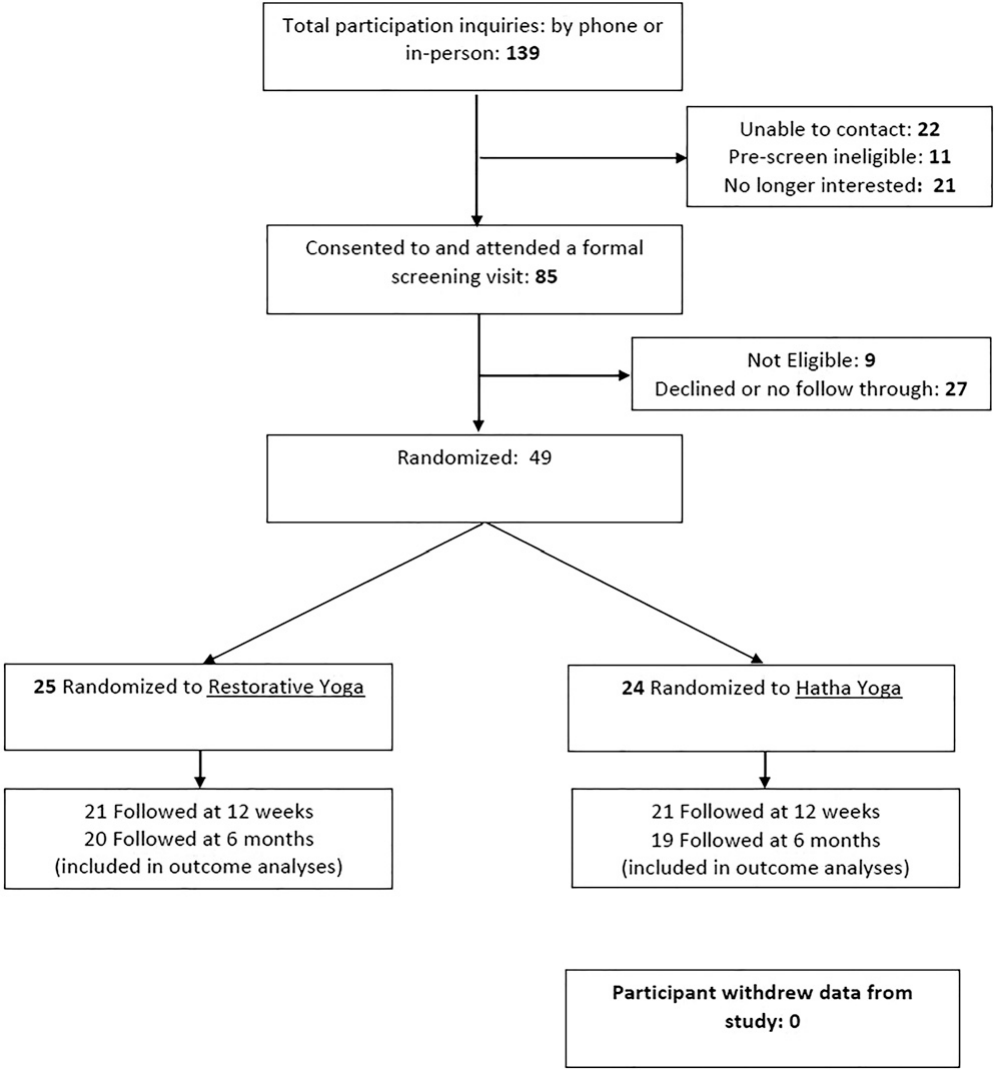
Randomized Trial Flowchart.

The study design included recruitment of 2 participant cohorts (target n = 25 each) for enrollment, assessments, and interventions. The pre-specified study recruitment goal was to enroll 50 participants within 12 months. although 18 months was the fesibility cutoff. The first cohort took just over 6 months to fill, while the second cohort was filled in 3-4 months, indicating strong feasibility. We enrolled 49 instead of 50 because of intervention timing and delays in the overall study timeline. The main change from cohort 1 to 2 was approval of credentials for study staff to recruit in person at the military medical facility. A total of 139 people were referred to or inquired about study participation over 10 months (See Figure 1). When contacted by telephone for pre-screening and to provide study information, 11 were not eligible, 21 were no longer interested and declined further screening, and 22 could not be reached. Subsequently, 85 individuals were scheduled for a formal screening and the informed consent process, at which 9 were found to be ineligible, primarily because of nerve compression or balance issues that were safety concerns. Of the 76 eligible participants, 49 attended a baseline assessment and were randomized to restorative yoga (n = 25) or hatha yoga (n = 24).

#### Retention

Feasibility of retention was evaluated against an a priori criterion of at least 75% of participants completing assessments at each assessment timepoint. The 12 weeks assessment was completed by 42 of 49 participants (86%) and the 6 month assessment was completed by 80% (39/49) of participants. One participant asked to be completely dropped from the study.

#### Attendance

The intervention consisted of both in-person attendance and daily home practice. The feasibility cutoff for in-person attendance was set at a minimum mean attendance of 50% of 12 sessions attended. Across all 49 participants randomized, mean attendance was 6.5 sessions or 54% of the 12 weekly sessions. As a proportion, 57% of all participants randomized attended at least 6 yoga sessions. However, when excluding those who never attended or participants who stopped attending because of adverse events, deployment or legal issues, mean attendance was 9.8 of 12 sessions (82%). A minimum level of yoga home practice was not pre-specified. The mean number of days per week of self-reported home practice was 2.0 days. Mean attendance was not significantly different between the 2 types of yoga but favored restorative yoga (6.7 sessions vs hatha - 6.3).

#### Adverse Events

No serious adverse events were reported by participants in the study. A total of 6 non-serious adverse events were reported (2 – hatha; 4 – restorative). Of the 6 adverse events, 4 were surgeries unrelated to yoga (2 in each group) that resulted in no further attendance of yoga classes. Surgeries were reported as 1- leg, 1- foot, 1 - back (due to prior condition), 1 – surgery type not reported. The 2 other non-serious AEs were a broken ankle incurred during sports and injury to shoulder and ankle from military training activity. Thus, no adverse events were linked to yoga participation.

#### Intervention Satisfaction

Program satisfaction rating are reported in Table 2. Over 90% of participants agreed or strongly agreed that they enjoyed participating, liked the instructor, and would like to continue doing yoga. Class duration, yoga as expected, and experiencing health benefits were also rated favorably. Social aspects of yoga were not targets of the interventions and had moderate ratings on average, as did the practical aspects of class times and study duration. There were no statistically significant differences between the 2 groups given the relatively small sample sizes. However, it was notable that mean ratings on a few variables (health benefits, yoga as expected, and class duration) were about .30 points higher in the restorative group.

**Table 2.** Program Satisfaction Ratings.

Program evaluation	Hatha yoga (mean, n = 20)	Restorative yoga (mean, n = 19)	% Agree or strongly agree	% Disagree or strongly disagree
Enjoyed participating	4.75	4.68	92.3	0
Liked instructors	4.65	4.79	92.3	0
Would like to continue yoga	4.70	4.68	92.3	0
Experienced health benefits	4.15	4.42	74.3	0
Class duration was sufficient	4.10	4.47	82.1	5.1
Yoga met expectations	4.10	4.42	80.0	0
Availability of class locations	4.00	4.15	68.3	9.8
Study length was sufficient	3.75	4.05	64.1	15.4
Common bond w/others	3.85	3.74	66.7	7.7
Availability of class times	3.75	3.70	58.5	24.4

### Health Outcomes

Given that our study was designed to test the feasibility and acceptability of this research and the yoga interventions in a military setting, the sample sizes do not provide sufficient statistical power to test hypotheses about intervention efficacy. Paired outcome data (baseline and 12 weeks) was available for 42 of 49 participants (86%) enrolled. Intent-to-treat analyses are presented in Table 3 for outcomes by style of yoga. Overall, small to moderate reductions were found for BPI pain severity, BPI pain interference, and PROMIS pain intensity. In addition, there were meaningful increases on scales of the SF12, grip strength, and balance. However, some of the effect sizes reflected little change or improvement. Table 4 provides data and pre-post effect sizes for per protocol analyses that focus on those attending at least 50% of intervention sessions/weeks. As expected, among the participants attending at least half of the yoga sessions, effects were larger and more robust across different outcome measures. In general, effects were diminished at 6 months.

**Table 3.** Health Outcomes (means [SD]; Intent to Treat Analyses).

Outcome	Hatha yoga (n = 21)			Restorative yoga (n = 21)				
	(Week 0)	(Week 12)	Mean difference (95% CI)	(Week 0)	(Week 12)	Mean difference (95% CI)	Pre-post Cohen’s d	
							Hatha	Rest
RMDQ	10.8 (5.2)	10.1 (6.3)	−.71 (−3.29; 1.86)	10.0 (5.6)	7.6 (6.1)	−2.38 (−4.62; −.14)	.12	.41
NDI	14.3 (9.3)	14.0 (10.7)	−.33 (−3.4; 2.7)	12.1 (9.0)	10.0 (8.9)	−2.10 (−4.94; .75)	.03	.24
BPI-PS	4.7 (1.7)	4.0 (2.3)	−.65 (−1.6; .25)	4.7 (1.8)	3.7 (1.9)	−.93 (−1.77; −.09)	.33	.50
BPI-PI	4.2 (2.2)	3.9 (2.4)	−.36 (−.51; 1.23)	4.0 (2.4)	3.2 (2.5)	−.82 (−1.69; .04)	.16	.34
PROMIS-PI	9.8 (1.7)	9.0 (2.4)	−.81 (−1.52; −.10)	9.5 (2.3)	8.6 (2.5)	−.95 (−2.12; .21)	.39	.40
SF12-PH	33.1 (7.3)	37.0 (8.1)	3.9 (1.01; 6.85)	36.5 (10.5)	39.2 (9.6)	2.78 (−.59; 6.14)	.51	.28
SF12-MH	47.1 (11.6)	45.2 (12.5)	−1.96 (−7.37; 3.46)	49.5 (9.5)	47.2 (10.6)	−2.39 (−7.73; 2.95)	−.16	−.24
FSS	4.0 (1.4)	4.4 (1.6)	.37 (−.29; 1.03)	4.0 (1.5)	3.8 (1.6)	−.16 (−.84; .53)	−.25	.10

Abbreviations: RMDQ, roland-morris disability questionnaire; NDI, neck disability index; BPI-PS, brief pain inventory-pain severity; BPI-PI, brief pain inventory-pain interference; PROMIS-PI, PROMIS-pain intensity; SF-12-PH, short-form health survey-physical health; SF-12-MH, short-form health survey-mental health; FSS, fatigue severity scale.

**Table 4.** Health Outcomes (means[SD]; Per Protocol Analyses).

Outcome	Hatha yoga (n = 11)			Restorative yoga (n = 15)				
	(Week 0)	(Week 12)	Mean difference (95% CI)	(Week 0)	(Week 12)	Mean difference (95% CI)	Pre-post Cohen’s d	
							Hatha	Rest
RMDQ	9.5 (2.9)	6.7 (3.8)	−2.73 (−4.633; −.83)	8.9 (6.0)	5.9 (5.7)	−3.00 (−5.52; −.43)	.81	.51
NDI	10.0 (8.7)	7.8 (5.8)	−2.18 (−6.94; 2.58)	12.2 (9.3)	11.0 (9.5)	−1.20 (−4.31; 1.91)	.30	.13
BPI-PS	4.1 (1.8)	2.8 (1.3)	−1.27 (−2.28; −.27)	4.3 (1.9)	3.4 (2.1)	−.87 (−2.03; .30)	.82	.43
BPI -PI	3.3 (2.1)	2.6 (1.8)	−.66 (−1.63; .31)	3.9 (2.7)	3.2 (2.9)	−.66 (−1.78; .46)	.34	.24
PROMIS-PI	8.7 (1.6)	7.7 (1.8)	−1.00 (−1.80; −.21)	9.1 (2.5)	8.5 (2.6)	−.60 (−1.90; .70)	.58	.23
SF12-PH	35.6 (7.5)	40.3 (7.8)	4.64 (.36; 8.92)	38.1 (11.0)	40.6 (9.5)	2.44 (−2.28; 7.16)	.61	.24
SF12-MH	48.4 (12.0)	51.2 (9.7)	2.82 (−2.56; 8.20)	47.5 (10.0)	47.8 (10.4)	.36 (−5.4; 6.12)	.26	.04
FSS	3.7 (1.5)	3.4 (1.1)	−.29 (−.95; .36)	4.2 (1.6)	3.8 (1.5)	−.44 (−1.29; .41)	.22	.29

Abbreviations: RMDQ, roland-morris disability questionnaire; NDI, neck disability index; BPI-PS, brief pain inventory-pain severity; BPI-PI, brief pain inventory-pain interference; PROMIS-PI, PROMIS-pain intensity; SF-12-PH, short-form health survey- physical health; SF-12-MH, short-form health survey-mental health; FSS, fatigue severity scale.

With our sample including military personnel with cLBP and/or cNP, we next looked at outcomes for those participants who only had back pain compared to those who either had only cNP or those that had both cNP and cLBP. As shown in Table 5, we found that the mean effect size across the most relevant measures reflected very little improvement among those with neck pain, whereas clinically meaningful moderate effects were found on average among those with cLBP only, despite slightly lower attendance. As expected, Supplemental Table 2 shows improvements among those attending 50% or more classes, with small effects emerging for those with cNP.

**Table 5.** Health Outcomes by Location of Pain.

Outcome	Back pain only (n = 21) Mean change (95% CI)	Neck pain (n = 21) Mean change (95% CI)
RMDQ	−2.81 (−5.07; −.55) (d=.57)	−.29 (−2.77; 2.20) (d = .05)
NDI	−1.62 (−5.02; 1.78) (d = .22)	−.81 (−3.31; 1.70) (d = .15)
BPI-PS	−1.27 (−2.05; −.49) (d = .74)	−.31 (−1.22; .60) (d = .15)
BPI -PI	−1.07 (−1.79; −.35) (d = .68)	−.11 (−1.06; .85) (d = .05)
PROMIS-PI	−1.33 (−2.30; −.36) (d = .62)	−.43 (−1.35; .49) (d = .21)
SF12-PH	4.93 (2.12; 7.73) (d = .80)	1.78 (−1.55; 5.10) (d = .24)
SF12-MH	−2.38 (−7.92; 3.16) (d = −.20)	−1.97 (−7.17; 3.24) (d = −.17)
FSS	.16 (−.39; .71) (d = −.13)	.05 (−.74; .85) (d = −.03)
Mean effect size - d	.41	.08
Sessions attended	5.9	7.0

Abbreviations: RMDQ, roland-morris disability questionnaire; NDI, neck disability index; BPI-PS, brief pain inventory-pain severity; BPI-PI, brief pain inventory-pain interference; PROMIS-PI, PROMIS-pain intensity; SF-12-PH, short-form health survey- physical health; SF-12-MH, short-form health survey-mental health; FSS, fatigue severity scale.

To further explore outcome effects or lack thereof, we examined outcomes for those with neck pain by separating them into the 2 styles of yoga to which they were assigned (Supplemental Table 3 and 4). We found that among participants with cNP only or both cNP and cLBP, hatha Yoga participants had lower attendance, minimal benefit on average (See Supplemental Table 2), and a few participants reported worsening neck pain and neck pain-related disability. The sample size is very small, but when looking at those who were able to attend regularly (Supplemental Table 4), moderate effects were present. Secondary outcomes are presented in Supplemental Table 5.

## Discussion

Our study results provide solid evidence that it is feasible to conduct a randomized controlled comparative effectiveness trial of yoga for cLBP and cNP among active-duty military personnel. Although the IRB process took longer than expected, it did not significantly delay the study. Participant recruitment and retention goals were easily met. Attendance was adequate but improvement in this area remains an ongoing goal. Both the hatha and restorative yoga interventions appear safe, with only minor adverse events reported, few of which appeared related to yoga. Finally, participants were highly satisfied with the yoga interventions themselves.

Participant recruitment started slowly, but as study staff adapted to requirements of recruitment at a military medical center, the second cohort of 25 people was recruited in less than 4 months. The overall sample was recruited in less than 10 months, suggesting a recruitment rate of 5 enrolled participants/month, but the rate was higher in the second cohort. The main change was gaining approved credentials for a study staff member to conduct research within the military medical facility. This approach is highly recommended for future studies because military health care providers or staff have many competing priorities that limit their ability to recruit for an outside study. However, military health staff were very welcoming and helpful with an external researcher present.

Retention was good at both the 12 weeks and 6 months assessments, 86% and 80% respectively. These rates are comparable to those of previous RCTs of yoga for military veterans^28^ and are well within the cutoffs recommended for avoiding bias in RCTs; at least 80% at the end of intervention (12 weeks in this study) and at least 70% at longer term follow-up assessments (6 months here).^61^ About 20% of the 6 month assessments could only be completed through an online survey. The online assessment process easily accommodated the self-reported outcome measures, but some secondary outcomes that involved physical performance tasks such as grip strength and balance could not be completed. In this sample, the primary reasons that in-person completion of assessments was not possible were military reassignment or deployment. Military reassignments or deployments often cannot be anticipated more than 1-3 months in advance and are a known challenge for conducting research in this population.

When looking at the mean attendance of in-person yoga sessions for the sample as a whole, 54% of sessions attended was sub-optimal and just surpassed the minimum cutoff of 50% of sessions attended. However, much of what appears as low attendance was the result of unrelated adverse events (n = 6) and deployment/reassignment (n = 3), as well as a few people who never attended (1 with legal problems, 2 that never responded). When excluding these 12 participants, the remaining 37 participants had a mean attendance of 82% of 12 sessions. Thus, this study identifies a different challenge beyond attendance once participants have started yoga. However, these data were obtained at 1 site in a pilot study with a relatively small sample size. Thus, it is unclear to what extent these challenges would generalize to other military research settings. To address these challenges with adherence in a larger study, investigators are considering additional exclusion criteria around whether surgeries are being considered, and the possibility of remotely delivered yoga sessions in the case of reassignment or deployment. Each type of yoga was offered at 3 different times per week, but with busy work schedules and many with family obligations, availability was still mentioned as an issue. Remotely delivered yoga can also help with scheduling challenges. Although the interventions in this study were completed prior to the COVID pandemic, remote delivery of yoga and other mind-body interventions became necessary during the pandemic in both research and non-research settings. Thus, experiences during the pandemic support wider use of remote intervention delivery.

The 6 adverse events reported were all unrelated to participation in yoga. Four of these events were reported by participants who never attended a class and included 3 surgeries and 1 sports-related broken ankle. Another participant injured their shoulder and ankle during military physical training and another had surgery for a broken foot (not sustained while performing yoga) near the end of the intervention period. Thus, the yoga interventions appeared quite safe for the participants enrolled. However, the outcomes analyses did reveal a possible trend toward increased pain and reduced function in some participants who had chronic neck pain and were assigned to the hatha yoga class. This finding was based on a very small sample and emerged only in the self-reported outcome assessments; it was not reported during monitoring of adverse events. Study investigators are actively working toward reviewing the hatha intervention and modifying it to address this issue.

Satisfaction with the intervention and with research participation was high. Items rated lower included more practical aspects of the study such as study length and the availability of class times and locations, despite classes being offered at 3 different times per week Thus, in planning for a larger study, we will explore whether it is possible to offer an additional class time, likely during a midday weekday option for participants who are temporarily not working. It was notable that the hatha yoga participants rated a few other aspects of the study lower, such as “experiencing health benefits” and “yoga met expectations.” When reviewing free text comments on these area, clear patterns did not emerge. Some participants were young and fit and expected yoga to be faster paced and more strenuous, while other participants had more serious chronic pain conditions that required a slower and safer style of yoga. As a result, the next study may benefit from focusing on 1 of these 2 subgroups as opposed to both.

Health outcome data suggest that both groups had small to moderate effect size improvements on a number of important outcomes including pain severity, disability, pain interference, and physical aspects of quality of life. Given the main goal of establishing feasibility for this research in military personnel with chronic pain, significant tests and/or conclusions about efficacy are not appropriate.

With intent-to-treat analyses, health outcome improvements were smaller than expected in some areas, possibly related to about 20% of the sample attending few or no intervention sessions. Other possible influences include the active duty military environment in which many participants were under stress and pressure to perform their occupational and family responsibilities, making it hard to prioritize their own health and well-being. To further explore our findings, we examined the change in outcomes for participants with cLBP only, vs those with only cNP or both cNP and cLBP. As shown in Table 4, we observe a pattern in which cNP participants reported very little health improvement on average. At the same time, improvement in those with cLBP alone was more robust and similar to findings in a previous study of yoga for cLBP^28^ even though attendance was slightly lower. When investigators adapted the previous intervention to include cNP, poses that could aggravate cNP were avoided or modified, but new poses were not specifically added. These data suggest that modifications to the intervention are needed to better address issues of cNP in military personnel.

A final analysis (Supplemental Tables 3 and 4) sought to examine any patterns by type of yoga in participants with cNP. With only about 10 participants in each group, data should be interpreted cautiously, but participants with cNP randomized to hatha yoga benefitted very little on average, with 4-5 of those participants reporting various increases in pain or functional limitations. However, when removing non-attenders, moderate effects were present. None of the participants reported non-attendance because they believed yoga increased their pain, but that remains possible. To address this concern, the intervention has been fully reviewed and a number of changes to the intervention are being tested in a separate study with military veterans with cNP and/or cLBP.

When comparing our results to the only other RCT of yoga for CLBP in military personnel,^36^ we find that attendance was lower in the current study, despite being quantified slightly differently. Highland et al compared individualized restorative yoga to a treatment as usual control group and found that about 85% of participants attended 75% (9 of 12) of the recommended yoga sessions. There are many differences between these studies that may contribute to these differences. Most notably, the Highland et al study used individualized yoga sessions which allows for scheduling at convenient times. This approach clearly has attendance benefits that may translate into better outcomes, but it may also be much more costly if implemented more widely. That study also included participants who were not active duty military, had an inactive treatment as usual control group, was conducted by military researchers, and examined outcomes with tests of statistical significance.

The current study has a number of limitations. The study was conducted, and feasibility was established, at a single site as a collaboration between academic researchers and healthcare providers at the Naval Medical Center San Diego. Thus, it is possible that the feasibility and acceptance results may not generalize to other military medical centers, geographical locations or to other yoga instructors. Some lessons learned may generalize to other military locations, but further feasibility work may be helpful at new research sites. The 2 types of yoga were mostly taught by different instructors; only 1 of 4 instructors taught both types of yoga. Thus, effects between types of yoga may be influenced by instructor characteristics. All instructors attended the same training session for either hatha or restorative yoga in an effort to minimize differences in instruction and standardize the intervention delivered.

Conducting this pragmatic study with persons with cLBP and/or cNP has led to a number of methodological challenges. Although attendance was adequate to establish feasibility and was similar to previous trials with military veterans,^28^ it was lower than in a previous study that used individualized yoga intervention appointments. Previous qualitative research suggested that providing yoga sessions at a military facility and providing greater availability were important issues.^23^ However, efforts in this study to provide early morning and early evening classes at the NMCSD and a weekend session in the community was still not enough to satisfy everyone’s schedule. Data also suggest that yoga may have increased pain in a subset of neck pain participants resulting in non-attendance. Second, it probably does not make sense to measure neck- or back-related disability in persons who do not report pain in those areas. At the same time, there is considerable overlap between these conditions. Thus, a larger future study may need to ensure an adequate sample size for subgroup analyses. Additionally, prior research suggests that stress and PTSD may exacerbate chronic pain conditions in military personnel.^16,19,20^ This study was not designed to measure or address these issues but they should be considered for the next study. Thoughtful planning about these and other issues are important for the success of a larger more definitive trial.

## Conclusion

Few other studies exist on the benefits of yoga for military personnel with cLBP and/or cNP. Results of this trial demonstrate the feasibility of conducting a randomized controlled comparative effectiveness trial of yoga for cLBP and cNP among active duty military personnel. Acceptability was also established for both styles of yoga. Additional work to enhance or alter the interventions for those with cNP appears warranted. Establishing feasibility at other military medical facilities will also help prepare for a fully-powered comparative effectiveness trial that may require more than a single site to ensure adequate sample size and better generalizability.

## Supplemental Material

Supplemental Material - Comparing Types of Yoga for Chronic Low Back and Neck Pain in Military Personnel: A Feasibility Randomized Controlled Trial Click here for additional data file. Supplemental Material for Comparing Types of Yoga for Chronic Low Back and Neck Pain in Military Personnel: A Feasibility Randomized Controlled Trial by Erik J Groessl, PhD, Danielle Casteel, MA, Symone McKinnon, MA, Adhana McCarthy, MPA, Laura Schmalzl, PhD, Douglas E Chang, MD, and Crystal L Park, PhD in Global Advances in Health and Medicine

## Appendix

### Abbreviations

QOL: Quality of Life
RCT: Randomized Controlled Trial
