# Supplementary material for: Comparing Types of Yoga for Chronic Low Back and Neck Pain in Military Personnel: A Feasibility Randomized Controlled Trial
Source: Glob Adv Health Med. 2022 Jun 16;11:2164957X221094596. doi: 10.1177/2164957X221094596 (PMC9208032; doi:10.1177/2164957X221094596)
Supplement: Supplemental Material - Comparing Types of Yoga for Chronic Low Back and Neck Pain in Military Personnel: A Feasibility Randomized Controlled Trial [file sj-pdf-1-gam-10.1177_2164957X221094596.pdf]

Supplemental Table 1. Inclusion / Exclusion Criteria and Method used to Determine Eligibility for Study Participation

**Inclusion Criteria**	**Determined by:**	**Exclusion Criteria**	**Determined by:**
Active-duty military employment and age $\geq$ 18	screening interview; medical record	back or neck surgery in the last 1 year	screening interview; medical record
Diagnosis of chronic LBP or neck pain > 6 months	screening interview; medical record	chronic pain due to specific systemic problem (e.g., lupus)	screening interview; medical record
Willing to attend 12 weeks of yoga	screening interview; informed consent	severe vertebral disk problems	screening interview; medical record
Willing to complete 2 assessments	screening interview; informed consent	persistent sciatica or nerve compression > 3 months	screening interview; medical record
English Literacy	screening interview; informed consent	coexisting chronic pain (migraines, fibromyalgia)	screening interview; medical record
No changes in pain treatments in the past month.	medical record; screening interview	Serious or unstable psychiatric illness (e.g. psychosis, mania)	screening interview; medical record
Willing to not change pain treatments unless medically necessary.	screening interview; informed consent	major coexisting medical illness (e.g., cancer, COPD, morbid obesity)	screening interview; medical record
Have not practiced yoga > 2x in the last 12 months	screening interview; informed consent	Positive Romberg test (with or without sensory neuropathy)	screening interview; medical record

Supplemental Table 2 - Health Outcomes by Location of Pain and attended at least 50% of classes

**Outcome**	**Back pain only (n=12)**<br>Mean change (95% CI)	**Neck pain (n=14)**<br>Mean change (95% CI)
RMDQ	-3.75 (-6.45; -1.05) (d=0.88)	-2.14 (-4.22; -0.07) (d=0.60)
NDI	-1.75 (-6.33; 2.83) (d=0.24)	-1.50 (-4.62; 1.62) (d=0.28)
BPI-PS	-1.56 (-2.67; -0.45) (d=0.89)	-0.6 (-1.67; 0.50) (d=0.31)

BPI -PI	-0.96 (-1.92; -0.01)<br>(d=0.64)	-0.40 (-1.54; 0.75)<br>(d=0.20)
PROMIS-PI	-1.00 (-1.77; -0.23)<br>(d=0.83)	-0.57 (-1.96; 0.82)<br>(d=0.24)
SF12-PH	4.84 (0.87; 8.81)<br>(d=0.78)	2.11 (-2.90; 7.11)<br>(d=0.24)
SF12-MH	1.00 (-3.71; 5.70)<br>(d=0.14)	1.75 (-4.64; 8.13)<br>(d=0.16)
FSS	0.03 (-0.69; 0.74)<br>(d= -0.03)	0.73 (-1.53; 0.07)<br>(d=0.53)
Mean effect size - d	**0.55**	**0.32**
Sessions attended	10.2	10.4

Supplemental Table 3 – Health Outcomes for Participants with chronic Neck Pain by Type of Yoga (intent to treat)

**Neck Pain**	**Hatha (n=11)**<br><br>Mean change (95% CI)	**Restorative(n=10)**<br><br>Mean change (95% CI)
RMDQ	1.4 (-2.80; 5.53)<br>(d= -0.22)	-2.10 (-4.99; 0.79)<br>(d=0.52)
NDI	-0.64 (-4.20; 2.92)<br>(d=0.12)	-1.0 (-5.29; 3.29)<br>(d=0.17)
BPI-PS	-0.14 (-1.47; 1.20)<br>(=0.07)	-0.50 (-2.02; 1.02<br>(d=0.24)
BPI-PI	0.18 (-1.30; 1.66)<br>(d= -.08)	-0.4 (-1.89; 1.04)<br>(d=0.21)
PROMIS-PI	-0.36 (-1.17; 0.45)<br>(d=0.30)	-0.50 (-2.45; 1.45)<br>(d=0.18)
SF12-PH	1.66 (-0.99; 4.30)<br>(d=0.42)	1.91 (-5.29; 9.10)<br>(d=0.19)
SF12-MH	-3.43 (-11.83; 4.97)<br>(d= 0-.27)	-0.35 (-7.91; 7.21)<br>(d= -0.03)
FSS	0.78 (-0.27; 1.83)<br>(d= -0.50)	-0.74 (-1.92; 0.43)<br>(d=0.45)

Mean effect size - d	**-0.02**	**0.24**
Sessions attended	6.1	7.8

RMDQ=Roland-Morris Disability Questionnaire; NDI=Neck Disability Index; BPI-PS= Brief Pain Inventory-Pain Severity; BPI-PI= Brief Pain Inventory-Pain Interference; PROMIS-PI = PROMIS-Pain Intensity; SF-12-PH= Short-form Health Survey- Physical Health; SF-12-MH= Short-form Health Survey-Mental Health; FSS= Fatigue Severity Scale.

Supplemental Table 4 - Health Outcomes for Participants with chronic Neck Pain by Type of Yoga (per protocol)

**Neck Pain**	**Hatha (n=5)** Mean change (95% CI)	**Restorative(n=9)** Mean change (95% CI)
RMDQ	-1.2 (-5.26; 2.86) (d=0.37)	-2.67 (-5.62; 0.29) (d=0.69)
NDI	-2.8 (-7.05; 1.45) (d=0.82)	-0.78 (-5.64; 4.08) (d=0.12)
BPI-PS	-0.75 (-2.16; 0.66) (d=0.66)	-0.50 (-2.22; 1.23) (d=0.22)
BPI-PI	-0.54 (-2.91; 1.82) (d=0.29)	-0.32 (-1.96; 1.33) (d=0.15)
PROMIS-PI	-0.60 (-2.48; 1.28) (d=0.40)	-0.56 (-2.77; 1.66) (d=0.19)
SF12-PH	2.36 (-2.70; 7.44) (d=0.58)	1.97 (-6.23; 10.16) (d=0.18)
SF12-MH	5.21 (-8.56; 18.97) (d=0.47)	-0.18 (-8.78; 8.43) (d=-0.02)
FSS	-0.33 (-1.43; 0.77) (d=0.38)	-0.95 (-2.18; 0.28) (d=0.59)
Mean effect size - d	**0.50**	**0.27**
Sessions attended	10.8	10.1

RMDQ=Roland-Morris Disability Questionnaire; NDI=Neck Disability Index; BPI-PS= Brief Pain Inventory-Pain Severity; BPI-PI= Brief Pain Inventory-Pain Interference; PROMIS-PI = PROMIS-Pain Intensity; SF-12-PH= Short-

form Health Survey- Physical Health; SF-12-MH= Short-form Health Survey-Mental Health; FSS= Fatigue Severity

	Hatha Yoga (n=21)			Restorative Yoga (n=21)		
**Outcome**	(Week 0)	(Week 12)	Mean difference (95% CI)	(Week 0)	(Week 12)	Mean difference (95% CI)
**Self-Efficacy**	5.7 (2.0)	5.3 (2.4)	-.39 (-1.49; 0.71)	5.9 (2.1)	6.0 (2.6)	0.33 (-0.98; 1.05)
**AUDIT-C**	2.2 (1.8)	1.9 (1.8)	-0.29 (-0.72; 0.15)	2.1 (1.6)	2.3 (1.5)	0.24 (-0.19; 0.67)
**Resilience (BRS)**	3.6 (0.8)	3.6 (0.7)	0.03	4.0 (0.8)	3.98 (0.9)	-0.02

Scale.

			(-0.26; 0.32)			(-0.30; 0.25)
**Depression (CESD 10)**	10.7 (5.4)	10.6 (6.3)	-0.05<br>(-2.34; 2.25)	8.7 (5.4)	9.8 (5.8)	1.10<br>(-2.0; 4.19)
**Anger (DAR)**	16.0 (13.3)	15.8 (15.0)	-0.14<br>(-5.51; 5.22)	13.6 (10.5)	14.4 (12.8)	0.81<br>(-3.45; 5.06)
	**Hatha Yoga (n=15)**			**Restorative Yoga (n=20)**		
**Balance – right, eyes open**	51.8s (15.7)	54.9s (14.8)	4.07<br>(-2.36; 10.49)	45.1s (20.2)	49.3s (18.7)	4.23<br>(-5.93; 14.38)
**Balance – left, eyes open**	51.5s (15.9)	59.9s (0.26)	7.87<br>(-0.41; 16.14)	49.8s (18.6)	48.1s (20.1)	-1.65<br>(-9.94; 6.64)
**Balance – right, eyes closed**	20.1s (18.1)	26.7s (20.3)	4.77<br>-2.18; 11.72)	11.6s (12.6)	18.9s (16.1)	7.33<br>(-0.89; 15.54)
**Balance – left, eyes closed**	22.7s (19.1)	15.1s (14.9)	-7.60<br>(-17.47; 2.27)	15.6s (13.7)	18.5s (17.8)	2.83<br>(-4.57; 10.22)
**Grip Strength - right**	88.8 (31.3)	94.6 (28.1)	5.83<br>(-0.84; 12.51)	83.5 (33.8)	88.6 (24.6)	5.08<br>(-2.72; 12.87)
**Grip Strength - left**	82.0 (28.8)	86.2 (25.2)	4.27<br>(-1.54; 10.07)	81.2 (29.8)	81.0 (26.8)	-0.25<br>(-3.69; 3.19)

Supplement Table 5 – Additional Secondary and Exploratory Outcomes

AUDIT-C=AUDIT-C 3-item; BRS=Brief Resiliency Scale; CESD 10= Center for Epidemiologic Studies Short Depression Scale 10-item; DAR= Dimensions of Anger Reactions
